# Uyghur–Chinese Adult Bilinguals’ Construal of Voluntary Motion Events

**DOI:** 10.3389/fpsyg.2022.892346

**Published:** 2022-07-14

**Authors:** Alimujiang Tusun

**Affiliations:** Pembroke College, University of Cambridge, Cambridge, United Kingdom

**Keywords:** Uyghur, Chinese, motion events, bilingualism, crosslinguistic influence

## Abstract

The study examined the implications of Talmy motion event typology and Slobin’s thinking-for-speaking hypothesis for the context of Uyghur–Chinese early successive bilingualism. Uyghur and Chinese represent genetically distant languages (Turkic vs. Sino-Tibetan) that nonetheless share important framing properties in the motion domain, i.e., verb-framing. This study thus aimed to establish how this structural overlap would inform bilingual speakers’ construal of motion events. Additionally, it sought to offer an “end state” perspective to a previous study on Uyghur–Chinese child bilinguals and to shed light on issues around the longevity of crosslinguistic influence. Thirty adult Uyghur–Chinese early successive bilinguals were invited to describe a set of voluntary motion events (e.g., “a man runs across the road”). Their verbalizations, alongside those from 24 monolingual Uyghur and 12 monolingual Chinese speakers were systematically analyzed with regard to the kind of linguistic devices used to encode key components of motion (main verb vs. other devices), the frequency with which the components are expressed together (Manner + Path) or separately (Path or Manner) and how they are syntactically packaged. The findings show that the bilinguals’ thinking-for-speaking patterns are largely language-specific, with little crosslinguistic influence. A comparison of our findings with previous studies on Uyghur-Chinese child bilinguals revealed no developmental change either in the analyzed aspects of motion descriptions or in patterns of crosslinguistic influence. As such, the findings lend support to accounts that propose crosslinguistic influence to be a developmental phenomenon.

## Introduction

How people think and talk about motion in space has served as a fruitful venue for examining the relationship between language and cognition. This study explored the implication of Talmy’s (2000) motion event typology and its subsequent articulations in relation to Slobin’s (1996, 2006) *thinking-for-speaking* hypothesis for the context of early successive bilingualism. While there are numerous studies in this regard (e.g., Hohenstein et al., 2006; Daller et al., 2011; Filipović, 2011; Engemann, 2021; Wang and Wei, 2021), they are mostly concerned with European languages, and as per Talmy’s typological classification, languages that are typological contrasting, i.e., verb-framed vs. satellite-framed (e.g., Spanish/French vs. English/German). We therefore know relatively little about what happens in language combinations beyond the Indo-European and that are typologically overlapping. The present study aims to bridge this gap by focusing on the bilingualism situation of an underrepresented Turkic language, i.e., Modern Uyghur (henceforth Uyghur) and a Sino-Tibetan language, i.e., Mandarin Chinese (henceforth Chinese). The two languages are genetically distant and yet they show important structural overlap in motion expression. As such, their combination is well placed to test some influential accounts of crosslinguistic influence (CLI) that emphasize the role of structural overlap. Furthermore, by focusing on Uyghur–Chinese adult bilinguals and comparing our findings with those of a previous developmental study on Uyghur–Chinese child bilinguals’ motion event construal (Tusun, 2019), the study aims to shed light on issues around the longevity of CLI (cf. Hulk, 2017; Serratrice, 2020).

## Motion Event Typology

Talmy (2000) observed that a motion event typically consists of a figure moving along a particular Path trajectory in a particular Manner with reference to a Ground. Within this macro-event, Path is said to be the framing event and Manner the co-event. And depending on where the framing event is expressed in a sentence, Talmy categorized the world’s languages into two major types. Thus, languages in which Path is expressed in the verb (root) are said to be verb-framed (e.g., Romance, Semitic, Japanese, Turkish) while those in which Path is expressed in a satellite, i.e., a constituent in construction with the main verb and syntactically subordinate to it as a dependent to a head (cf. Talmy, 2016), are argued to be satellite-framed (e.g., Germanic languages). In the English example (1), Manner is expressed in the verb and Path in a satellite whereas in Spanish, as in (2), main verb expresses Path while Manner is expressed in an adjunct. However, other scholars pointed out that satellite-framed constructions are also licensed in verb-framed languages insofar as a given event does not involve crossing a spatial boundary, a phenomenon known as the boundary-crossing constraint (Aske, 1989; Slobin and Hoiting, 1994). In (3), Manner is expressed in the verb, but Path (i.e., Goal or the Vector dimension of Path in Talmy’s terminology) is expressed in a satellite.


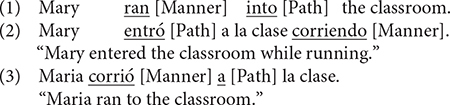


The implications of Talmy’s seminal typology for spatial language use has been most extensively examined in relation to Slobin’s *thinking-for-speaking* hypothesis (e.g., Slobin, 1996, 2003, 2004, 2006; Daller et al., 2011; Filipović, 2011; von Stutterheim et al., 2017, 2020; Wang and Wei, 2021). And a consistent observation has been that speakers of satellite-framed languages typically produce semantically richer or denser descriptions (mentioning both Path and Manner) while speakers of verb-framed languages tend to produce semantically less rich utterances, focusing primarily on Path (e.g., Slobin, 2004; Özçalışkan, 2015; Hickmann et al., 2018; Tusun and Hendriks, 2019; Bunger et al., 2021; Hendriks et al., 2021). The *thinking-for-speaking* hypothesis postulates that speakers of typologically distinct languages may not necessarily perceive or conceptualize a given event differently. However, for the purpose of verbalizing that event, they are obliged to fit their conceptualization into the most typical ways in which event components are lexicalized and syntactically packaged in their language. Going back to the typological contrast illustrated in (1) to (3), speakers of satellite-framed languages have at their disposal compact constructions (mono-clausal) that facilitate the simultaneous expression of Manner and Path. However, speakers of verb-framed languages typically have to employ complex constructions (bi-clausal with subordination) to achieve the same effect (cf. Allen et al., 2007; Harr, 2012; Hendriks et al., 2021) and since doing so may increase online processing load (cf. Özçalışkan and Slobin, 2003; Slobin, 2004; Özçalışkan, 2015), unless the co-event is at issue, speakers typically leave it unspecified and focus on the framing event itself. The sustained use of such language-specific patterns and by implication, the habitual exercise in language-specific ways of formulating pre-verbal message, i.e., *thinking-for-speaking* (cf. Levelt, 1996; von Stutterheim and Nüse, 2003), renders Manner a highly codable domain for speakers of satellite-framed languages but not necessarily for verb-framed languages. This is argued to explain the point made earlier about why speakers also differ along typological lines in the semantic density of their motion descriptions.

## Motion Event Expressions in Bilingualism

Motion event typology has served as an extremely useful framework for examining bilingual cognition and language use. Two issues that have been at the heart of much research is whether and to what extent bilinguals develop language-specific patterns of *thinking-for-speaking* and the role of CLI (e.g., Hohenstein et al., 2006; Daller et al., 2011; Engemann et al., 2012; Aveledo and Athanasopoulos, 2016; Engemann, 2016, 2021; Miller et al., 2018; Wang and Wei, 2021). The studies have mostly focused on typologically contrasting languages (English vs. French/Spanish) and have involved both child and adult bilinguals. A recurrent observation has been that, while bilinguals generally think for speaking in language-specific ways, their motion descriptions are characterized by CLI. Thus, bilingual speakers are found to use more Path verbs in their satellite-framed language and more Manner verbs in their verb-framed language (cf. Hohenstein et al., 2006; Engemann, 2013; Engemann, 2016; Aveledo and Athanasopoulos, 2016; Miller et al., 2018; Park, 2020). There is also some evidence suggesting that CLI extends to the verbal periphery. In terms of Manner expression, for example, Spanish-English bilinguals were found to use less Manner modifiers in Spanish (L2 to L1 influence) and in English (L1 to L2 influence) (Hohenstein et al., 2006). As to Path expression, Engemann (2013, 2016) reported that English-French bilingual children consistently express Path in peripheral devices in their French due to influence from English (also see Engemann et al., 2012). Additionally, while the issue regarding the longevity of CLI remains largely unexplored (cf. Serratrice, 2016), a couple of extant studies indicate that CLI may increase over time (cf. Aveledo and Athanasopoulos, 2016; Engemann, 2021).

A number of factors have been proposed to underlie CLI. In terms of language-internal factors, structural overlap seems to be a prime candidate (e.g., Hohenstein et al., 2006; Filipović, 2011; Miller et al., 2018; Park, 2020). With regard to the impact of language-external factors, bilingual speakers’ lexicalization patterns have been shown to reflect the patterns characteristic of the societally dominant language (Hohenstein et al., 2006; Daller et al., 2011). So language dominance is a key factor. Furthermore, going by the earlier observation that CLI seem to increase over time, and using age as a proxy for relative proficiency, it seems proficiency is another factor (cf. Aveledo and Athanasopoulos, 2016; Engemann, 2021). Finally, relative systematicity of the target systems seem to be an important factor as well. For instance, in studying how English-French bilingual children describe caused motion events (e.g., “A man pushed a pram across the road”), Engemann et al. (2012) observed highly persistent English to French CLI, which, these scholars attribute to the relative systematicity that the two languages display in the motion domain: English has a transparent system (i.e., Manner in the verb and Path in the satellite) while the French system shows considerable variability. It seems then that CLI goes from the more transparent system to the less transparent one. Note, finally, that, given that the same child bilinguals displayed little sign of CLI when describing voluntary motion (e.g., “A bear climbed up the tree”) (Engemann, 2021), a presumably less complex event type (i.e., fewer semantic components – Manner, Path) compared to caused motion (i.e., multiple semantic components – Manner, Path, Cause), it is likely that the magnitude and longevity of CLI interact with the relative task complexity.

## Uyghur and Chinese in Motion Event Typology

Before delving into motion expressions in Uyghur, a few words should be said about the Uyghur language itself. Uyghur belongs to the South-Eastern branch of the Turkic language family. It is, alongside Uzbek, the direct descendent of Chaghatay Turkic, which was the transregional literary of Islamic Central Asia until early 20th century (cf. Boeschoten and Vandamme, 1998). It is primarily spoken in China’s Xinjiang Uyghur Autonomous Region (XUAR) and is co-official with Chinese. It has more than 11 million speakers in XUAR (Memtimin, 2016) and another half a million in diaspora communities in Central Asian countries (Johanson, 2009). It is written with a reformed version of the Arabic alphabet. In terms of its general typological profile, Uyghur is an agglutinative language with an SOV word order. Its syntax is left-branching and modifiers precede their heads. It primarily displays suffix-based morphology where stems and suffixes are subject to sound harmony (Ragagnin, 2018).

In terms of motion expressions in Uyghur, Talmy (2000) categorized Turkish and by extension all Turkic languages as verb-framed. Example (4) illustrates a typical motion construction in Uyghur that is indeed verb-framed: Path expressed in the main verb and Manner in the subordinate clause *via* a converb, the functional equivalent of gerunds in European languages (cf. Johanson, 2021). Several recent studies examining the typological status of Uyghur do show that it is a typical verb-framed language. For instance, Tusun and Hendriks (2019) found that, when asked to narrate animated cartoons that featured both human and animal agents moving along different path trajectories, Uyghur speakers typically encoded Path in the main verb and Manner (when expressed) in the converb. Uyghur speakers also made abundant use of case marking to provide additional Path information (Source, Goal). Tusun and Hendriks (in press) observed the same systematicity in the verb and the verbal periphery in the context of caused motion as well (e.g., “A man pushed a box up the hill”). However, Tusun (in press) notes that, while Uyghur is predominantly verb-framed, it also licenses satellite-framed constructions, as in (5), when no boundary-crossing is involved (cf. Aske, 1989; Slobin and Hoiting, 1994; Özçalışkan, 2015).


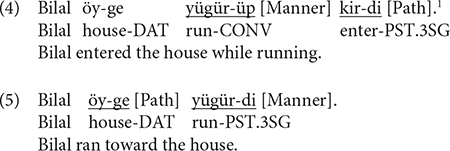


The status of Chinese in motion event typology has been a topic of much debate. Talmy (2000) initially categorized Chinese as a satellite-framed language. Example (6) represents a motion construction in Chinese where Manner and Path are, respectively, encoded in the V1 and V2 slot of a resultative verb compound (RVC). Talmy observed that the V2 element in the RVC constitutes a closed class of items and that it is where semantic categories such as aspect and resulting state are expressed, much like the directional particles in Germanic languages. He thus considered the Path-encoding morphemes in the RVC as satellites. However, other scholars (e.g., Slobin, 2004) noted that, given the absence of morphological marking in Chinese, it is hard to determine the main verb vs. the satellite in an RVC. These scholars proposed that Chinese, alongside other serial verb languages, should constitute its own category of equipollently framed languages. Moreover, as exemplified in (7), the V2 element in an RVC can function as full verbs in Chinese, which is essentially different from the Path particles in European languages. Note further that in such verb-framed constructions, Manner is typically expressed in a subordinate/adverbial clause marked by the durative aspectual marker *zhe*), which is, in the context of expressing motion, functionally equivalent to gerunds in European languages (cf. Ji et al., 2011b) and converbs in Turkic languages (cf. Johanson, 2021).


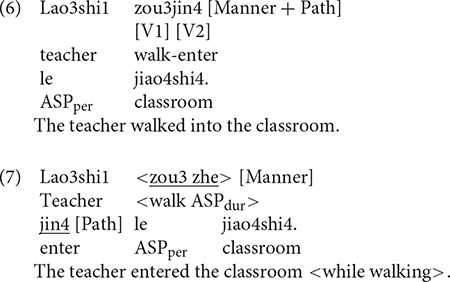


Recently, Talmy (2016) concedes that constructions like (6) where the Path-encoding morpheme in an RVC can function as an independent verb, are indeed equipollently framed. And recent studies on Chinese speakers’ non-verbal cognition seem to support this. For example, in a study on how English and Chinese speakers categorize motion events, Ji and Hohenstein (2018) found that, while English speakers were Manner-oriented, Chinese speakers were equally Manner- and Path-oriented. These authors explain differences across the two languages in language-specific terms: English, as a satellite-framed language, expresses Manner in the verb whereas Chinese, as an equipollently framed language, encodes Manner and Path in linguistic devices that share equal grammatical status. Further evidence for the equipollence of Chinese comes from studies on co-speech gestures which show that, while English speakers tend to highlight Manner in their gestures, Chinese speakers tend to highlight both Manner and Path (cf. Brown and Chen, 2013; Brown, 2015). Other more linguistically oriented studies have established that, beyond the RVC constructions, verb-framed constructions like (7) constitute a characteristic strategy for encoding motion in Chinese (cf. Ji et al., 2011b; Ji and Hohenstein, 2014; Shi et al., 2018). That is, despite that Uyghur and Chinese belong to two genetically distant language families, they share a key lexicalization pattern in expressing motion, i.e., the verb-framing pattern.

## Accounting for Crosslinguistic Influence: Structural Overlap and Co-Activation

In section “Motion Event Expressions in Bilingualism,” I touched upon several factors identified as giving rise to CLI. To explain CLI in a principled way, however, we need to look at more general accounts of CLI and in this study, I will focus on two specific accounts: structural overlap and co-activation.

The structural overlap hypothesis (Hulk and Müller, 2000; Hulk, 2017) posits two specific conditions for CLI to occur. First, language A allows only one option for a particular structure that (partially) overlaps with one of multiple structural options available in language B. Second, the structure at issue is at the interface between modules of grammar, and more particularly between pragmatics and syntax. The two conditions were presumed to be necessary but not sufficient, but when they are met, CLI was expected to occur in the shape of an overuse or overreliance on the shared structure in language B for an extended period of time. CLI is therefore expected to be quantitative and unidirectional, from language A to language B. In terms of its longevity, CLI is expected to be a developmental phenomenon that phases out over time. The structural overlap hypothesis has since gained considerable support from studies involving language pairs that fulfilled two conditions (e.g., Müller and Hulk, 2001; Schmitz et al., 2012). But other research also showed that the hypothesis can account for phenomena at the syntax-semantics interface (e.g., Serratrice et al., 2009; Liceras et al., 2012) as well as for issues that do not concern any interface (e.g., Foroodi-Nejad and Paradis, 2009; Nicoladis et al., 2010; Engemann et al., 2012; Bosch and Unsworth, 2020).

The co-activation account (cf. Nicoladis, 2006; Nicoladis et al., 2010; Nicoladis and Gavrila, 2015) essentially views CLI as an epiphenomenon of the simultaneous activation of the bilinguals’ languages during online production. Assuming that speech production is a staged process (e.g., Levelt et al., 1999), speakers start out by formulating the message they intend to express (i.e., the conceptualization stage) and then access the relevant grammatical/morphological/lexical frame (i.e., the lemma stage) and finally the corresponding phonology. In the case of bilingual speakers, due to the constant co-activation of both languages, the relevant language-specific structures compete for realization at the lemma stage. And while the structure in the target language enjoys greater level of activation, the one in the non-selected language occasionally wins out, likely due to recent exposure or language use, thereby giving rise to CLI. Note that Nicoladis (2006) speculation that recent exposure may induce CLI is compatible with findings from crosslinguistic priming research that bilinguals can even be primed to produce ungrammatical (Hsin et al., 2013) or discourse-pragmatically sub-optimal constructions (Hervé et al., 2016). Indeed, this new understanding of CLI as reconceptualized within the framework of crosslinguistic priming has gained support in some recent developmental studies (e.g., Hervé and Serratrice, 2018; Engemann, 2021).

In terms of its predictions, the co-activation account would expect CLI, understood as a by-product of crosslinguistic priming, to be bidirectional, given that the dominant structure in language A would co-activate the one in language B. Furthermore, CLI can occur both quantitatively, when there are overlapping options and qualitatively in the absence thereof. As to its longevity, two predictions can be entertained. On one hand, it can remain constant across the developmental span given that co-activation is a general bilingual phenomenon. On the other, it is possible that CLI increases with time, because more advanced language skills in the two languages may allow for more structures to be co-activated (cf. Bernolet and Hartsuiker, 2018; Van Gompel and Arai, 2018; Bosch and Unsworth, 2020).

## The Case for Studying Motion Event Expressions in Uyghur–Chinese Adult Bilinguals

So what does studying Uyghur–Chinese adult bilinguals’ expression of motion events add to our current knowledge? In addition to the obvious merit of diversifying the bilingualism research landscape by introducing two underrepresented languages, the gaps this study aims to bridge are much more specific. Firstly, it has become clear by now that most of the studies in this domain have focused on typologically contrasting languages, i.e., verb-framed vs. satellite-framed, and on Indo-European languages. As detailed in section “Uyghur and Chinese in Motion Event Typology,” Uyghur and Chinese, despite having distinct typological profiles (verb-framing vs. equipollent framing) display a clear structural overlap, i.e., verb-framing. As such, this language pair will help us better delineate the role of structural overlap in CLI as predicted by the structural overlap and co-activation accounts. Secondly, beyond motion event typology, the two languages represent distinct language families (Turkic vs. Sino-Tibetan) that differ vastly in their morphosyntactic systems. As such, their combination extends existing research that has predominantly involved Indo-European languages and can help us better understand whether the relative typological distance and differences across languages can differentially impact the likelihood and the extent of CLI in bilinguals (e.g., Filipović and Hawkins, 2019; Bassetti and Filipović, 2021).

Additionally, the findings of this study will shed light on the under-researched topic of the longevity of CLI in the motion domain (cf. Hulk, 2017; Serratrice, 2020). In Tusun (2019), I investigated how 4-, 6-, 8-, and 10-year-old early successive bilinguals (with an age of onset of 3;2) acquired motion expressions in their L1 Uyghur and L2 Chinese. A striking finding from that study concerned the child bilinguals’ development in their L2 Chinese in that, while monolingual Chinese children were fully adult-like in using the equipollently framed pattern and the verb-framed pattern from 3 years of age, the child bilinguals did so only at age 10, while relying on the verb-framed pattern as a dominant strategy until age 8. I attributed the bilinguals’ distinct developmental trajectory to the impact of CLI and concluded that CLI could be a developmental phenomenon. Using the same experimental paradigm and focusing on adult bilinguals who have very similar profiles as the child bilinguals in Tusun (2019) (see the next section), this study offers an “end-state” perspective on motion event expression in Uyghur–Chinese early successive bilingualism. As such, its findings will allow for an indirect testing of the predictions the structural overlap hypothesis and the co-activation account make for the longevity of CLI.

### Research Questions and Predictions

This study was motivated by two main research questions: (1) How do Uyghur-Chinese adult bilinguals construe voluntary motion events compared to monolingual controls? (2) Whether and to what extent the bilinguals’ descriptions are shaped by crosslinguistic influence?

To answer these questions, I focused on three aspects of the speakers’ motion verbalizations: (1) Information Locus examined the types of semantic components encoded in the main verb and in other devices; (2) Utterance Density captured the number of semantic components speakers expressed within a motion construction, for which two levels of density were established: utterance density 1 (UD1) if only one component was expressed (either Manner or Path) and utterance density 2 (UD2) if both components were expressed; and (3) Syntactic Packaging measured how the semantic components were packaged within a sentence, for which two categories were distinguished: Tight-Simple if Manner and/or Path were expressed in a single clause and Tight-Complex if they were distributed across two clauses *via* subordination (see details in section “Results” below).

Based on the literature and the two accounts of CLI, I made the following predictions. For Uyghur, I predicted the bilinguals to follow the monolingual pattern in encoding Path in the main verb and Manner (when expressed) in the converb. Besides, the bilinguals were predicted to provide extra Path information *via* case marking. Since the experimental stimuli involved events with rather clear Path and Manner dimensions, I expected the bilinguals, like the monolinguals, to predominantly express UD2 utterances couched in Tight-Complex constructions. For Chinese, the bilinguals were predicted to follow the equipollently framed Chinese pattern of simultaneously encoding Path and Manner in the RVC. Consequently, they would predominantly produce UD2 utterances in Tight-Simple constructions. However, in light of the structural overlap hypothesis and the co-activation account, I predicted some degree of unidirectional CLI from Uyghur to Chinese such that the bilinguals would show a greater tendency to use verb-framed constructions in their L2 Chinese, compared to monolinguals. Specifically, they would express only Path in the verb locus, and Manner in a subordinate clause, which would give rise to a more frequent use of Tight-Complex constructions.

### Participants

The participants comprised three groups: 30 Uyghur–Chinese adult bilinguals, 24 monolingual Uyghur speakers, and 12 monolingual Chinese speakers. The bilinguals were first-year university students in China and following Tusun (2019), a language background questionnaire was administered where only those who had fulfilled the following essential criteria were invited for participation. They were born to Uyghur parents, spoke Uyghur at home and had their first exposure to Chinese from around age 3–4 at kindergarten and later went to Chinese-medium schools throughout their education up to the university. They used Chinese at school and Uyghur outside school on a daily basis and the difference in their self-rated fluency in the two languages did not diverge (on a scale from 1 to 10) by more than two points. The monolingual Uyghur speakers were first-year university students in XUAR. Note that they had had some formal learning of Chinese since middle school but reported rather low proficiency in the language. They were therefore not “pure” monolinguals, but “pure” monolinguals are hard to come by in XUAR due to its widespread bilingualism. Rather, their language profiles were reflective of Uyghurs living in XUAR (see Li, 2020 for a recent survey) and these speakers were considered “monolingual” for the present purposes. Chinese monolinguals were university students in Beijing (see Ji et al., 2011a).^2^

### Experimental Stimuli and Procedure

The experimental stimuli consisted of a set of 18 short animated cartoons in which a protagonist moved along either a vertical (UP/DOWN) or boundary-crossing paths (ACROSS) in a particular manner (see Appendix 1 for an example).^3^ Each path type was represented 6 times, making a total of 18 experimental items (see Appendix 2 for a summary). They were randomized into six test orders and were assigned to the participants randomly. The monolinguals performed the task either in Uyghur or in Chinese. The bilinguals performed the task twice, once in Uyghur and once in Chinese, and to minimize task repetition effects, half of the bilinguals performed the task first in Uyghur and the other half first in Chinese. The interval between the two experimental sessions for the bilingual participants was about 1–2 weeks. To maximally induce a monolingual mode, the bilingual participants were interviewed by an Uyghur interlocutor for the Uyghur session and a Chinese interlocutor for the Chinese session.

Participants were met individually in a quiet room and the stimuli were presented on a computer screen. To ensure that they maximally relied on linguistic means (rather than pointing or other gestures), they were instructed to narrate what they had watched to an imaginary figure who had no visual access to the cartoons but who would have to reconstruct their content based on the participants’ descriptions. To familiarize the participants with the task, each session started with a training item. In most cases, they would mention both manipulated components, i.e., Manner and Path, but when they occasionally failed to do so, probes were made so that they would at least notice the two components. No such prompts were given for the experimental items.

### Coding

Examples (8) to (11) illustrate the kind of responses elicited, which, as can be seen, differ in terms of the kind of semantic components expressed and where they are expressed in the sentence. For example, in (8), Manner is expressed in the main verb while the entire event is construed as locative, i.e., no Path trajectory. In (9), which is a verb-framed construction, Path is expressed in the main verb and Manner in the subordinate/converbial clause. In (10), Manner and Path are expressed in an RVC, which is equipollently framed. In (11), which is a verb-framed construction, Path is expressed in the main verb and Manner in the adverbial clause. Each response was segmented into clauses, with a clause defined as a unit containing one verb and its arguments (cf. Hendriks et al., 2021). Thus, responses such as (8) and (10) were taken as consisting of one clause while (9) and (11) were segmented into two clauses, i.e., the matrix clause, indicated as (c2) and the converbial/adverbial clause as the subordinate clause, marked as (c1). Each clause was then coded in terms of the number and types of semantic components expressed (e.g., Manner-only, Path-only, Manner + Path), how they were encoded within the clause (e.g., main verb vs. other devices/satellite) or across clauses (with or without subordination). In line with Talmy (2016) and Ji and Hohenstein (2018), both V1 and V2 elements of the RVC were treated as verbs and coded as such. Following Croft et al. (2010) and Levin and Rappaport Hovav (2019), an expanded notion of the satellite was adopted to include any device except the verb that encodes spatial information. Thus, dative and ablative case markers, postpositions and converbs in Uyghur and nominal phrases, prepositions and adverbials in Chinese were considered satellites.


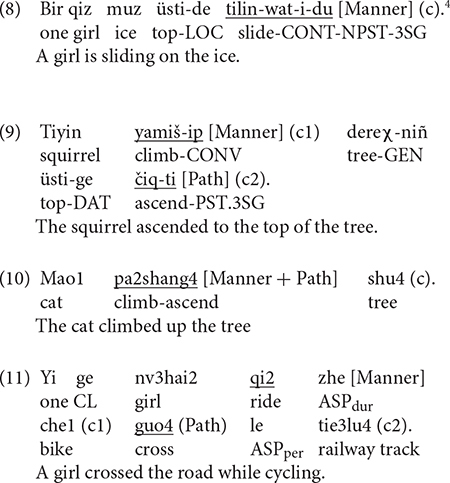


### Analyses

For our quantitative analyses, speaker group and path type were considered the independent variables whereas the different categories of the continuous dependent variables were (a) Path in the verb, Manner in the verb, Manner + Path in the verb; (b) Path in the satellite, Manner in the satellite, Manner + Path in the satellite; (c) UD1 responses, UD2 responses; and (d) Tight-Simple strategy, Tight-Complex strategy. The count data were analyzed by fitting generalized linear mixed-effect models with Poisson distribution, using R (R Core Team, 2017, glmer () function). For all models fitted, I included random intercepts for participant and path type. I first fitted a model including the main factors of interest and then fitted a reduced model excluding one of the factors to the same data. I followed this by comparing the relative goodness of fit of the two models using a likelihood ratio test *via* the anova() command, which revealed the relative fits (expressed as log likelihood) of the two models to test the statistical significance of the factor removed in the reduced model. By using Tukey multiple pairwise comparisons *post hoc* tests, I recorded the Chi-square statistics, degrees of freedom and *p*-value for the tests.

## Results

### Information in the Verb Locus in Uyghur

As mentioned above, this measure focused on the sorts of semantic components expressed in the main verb. In Uyghur, there were only two possibilities: Path in the verb, as in (12) or Manner in the verb, as in (13).


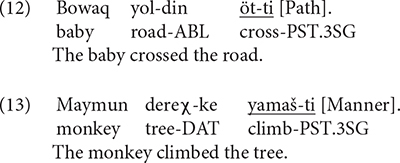


**FIGURE 1:**
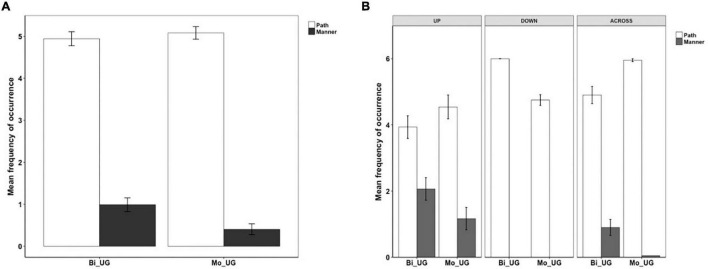
Information in the verb locus in Uyghur.

Figure 1A below shows information expressed in the verb locus. As we can see, both bilinguals (Bi_UG) and monolinguals (Mo_UG) predominantly encoded Path in the verb whereas expressing Manner in this locus was rather infrequent. This difference between the two categories within each group was significant [Bi_UG: χ^2^(1) = 275.52, *p* < 0.001; Mo_UG: χ^2^(1) = 340.3, *p* < 0.001]. A two-way packaging (Path, Manner) × group (Bi_UG, Mo_UG) analysis revealed a significant interaction [χ^2^(1) = 15.729] because the bilinguals encoded Manner more frequently than monolinguals [χ^2^(1) = 5.5956, *p* = 0.018]. No difference was found between the groups for Path. Figure 1B depicts information in the verb as a function of path type. As is clear, the general distribution of the two patterns mirrors the overall pattern as seen in Figure 1A, although Manner in the verb occurred for UP and ACROSS events only. A series of two-way packaging (Path, Manner) × group (Bi_UG, Mo_UG) on the three path types revealed significant interactions for UP [χ^2^(1) = 7.675, *p* = 0.005] and ACROSS [χ^2^(1) = 27.409, *p* < 0.001] events due to the bilinguals’ consistently more frequent expression of Manner than monolinguals [UP: χ^2^(1) = 3.7393, *p* = 0.053] and ACROSS [χ^2^(1) = 7.4298, *p* = 0.006] events.

### Information in the Verb Locus in Chinese

For Chinese, three categories were established. In (14), Path is expressed in a serial verb. In (15), the verb carried Manner information and in (16), Manner and Path are simultaneously expressed in an RVC.


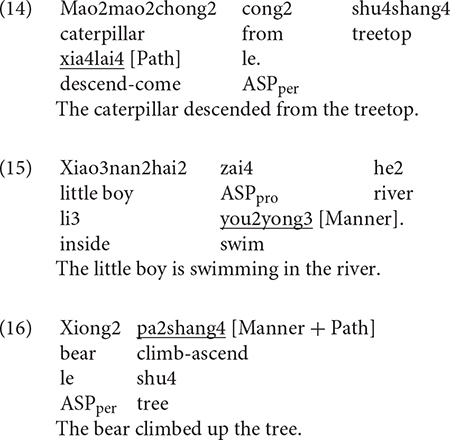


**FIGURE 2:**
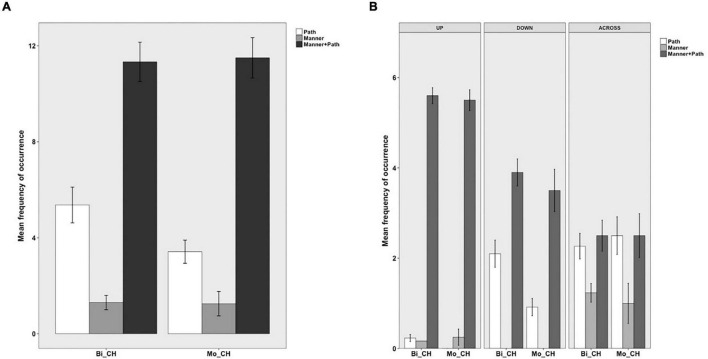
Information in the verb locus in Chinese.

Figure 2A below illustrates information expressed in the verb locus in Chinese. As we can see, the bilinguals (Bi_CH), like the monolinguals (Mo_CH), primarily expressed Manner + Path in the verb locus, which was followed by encoding Path and then Manner. The differences amongst the three categories within each group was significant [Bi_CH: χ^2^(1) = 277.26, *p* < 0.001; Mo_CH: χ^2^(1) = 128.01, *p* < 0.001]. A two-way packaging (Path, Manner, Manner + Path) × group (Bi_CH, Mo_CH) interaction analysis was not significant, meaning that the distribution of the three strategies did not vary by group. Figure 2B shows information expressed in the verb locus in Chinese as a function of path type. A series of two-way packaging (Path, Manner, Manner + Path) × group (Bi_CH, Mo_CH) interaction analyses on the UP, DOWN, and ACROSS events did not show significance. This indicates that the lack of difference across the two groups, as observed in the overall analysis, also held for each path type. However, two points should be highlighted here. First, it is remarkable that the bilinguals displayed the same sensitivity that monolinguals did toward the differing encoding strategies relative to different path types: both groups predominantly expressed Manner + Path for UP and DOWN events while their use of the three patterns was more varied for ACROSS events. Second, the bilinguals very occasionally (7 instances) expressed Path for UP events whereas the monolinguals did not.

### Information in the OTHER Locus in Uyghur

Four categories were established for information expressed in the OTHER locus. “Path” if, as in (17), additional Path information (i.e., Goal) is provided *via* a case marker; “Manner,” as in (18) where only Manner information is expressed in a converb without any further spatial information; “Manner + Path” if, as in (19), the OTHER locus contained Path information (i.e., Ground) and Manner in a converb; “Zero” if the response is a bare verb construction, as in (20).


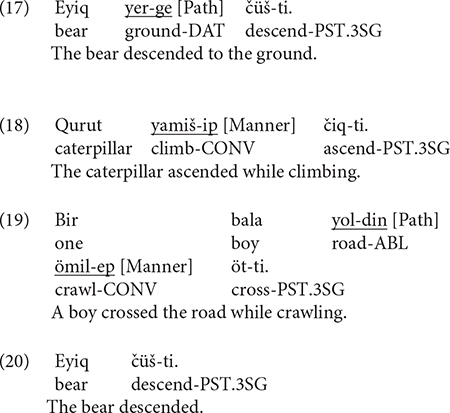


**FIGURE 3:**
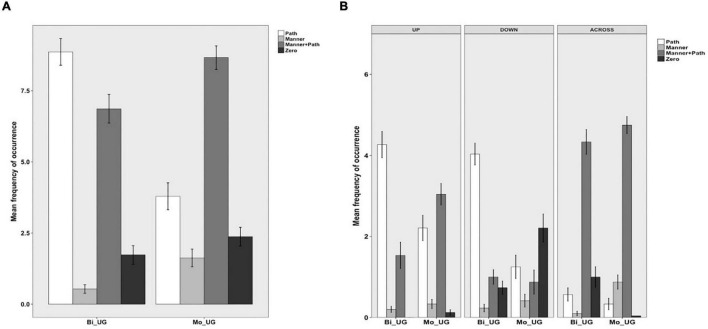
Information in the OTHER locus in Uyghur.

Figure 3A depicts information in the OTHER locus in Uyghur. The differences amongst the four patterns were significant for both bilinguals [χ^2^(3) = 367.46, *p* < 0.001] and monolinguals [χ^2^(3) = 159.91, *p* < 0.001]. A two-way packaging (Path, Manner, Manner + Path, Zero) × group (Bi_UG, Mo_UG) analysis revealed a significant interaction [χ^2^(3) = 77.182, *p* < 0.001] because, while bilinguals expressed Path more than monolinguals [χ^2^(1) = 8.6845, *p* = 0.003], they expressed Manner and Manner + Path less frequently than the latter [χ^2^(1) = 5.9519, *p* = 0.014; χ^2^(1) = 4.0616, *p* = 0.043, respectively]. Figure 3B displays information expressed in the OTHER locus in Uyghur as a function of path type. A series of two-way packaging (Path, Manner, Manner + Path, Zero) × group (Bi_UG, Mo_UG) analyses on the three path types revealed significant interaction for UP [χ^2^(3) = 36.809, *p* < 0.001], DOWN [χ^2^(3) = 59.156, *p* < 0.001], and ACROSS [χ^2^(3) = 49.681, *p* < 0.001] events, due to the following differences. For UP events, bilinguals expressed Path more frequently [χ^2^(1) = 6.4031, *p* = 0.011] but Manner + Path and Zero less frequently than monolinguals [χ^2^(1) = 5.6432, *p* = 0.017; χ^2^(1) = 4.2706, *p* = 0.038, respectively]. For DOWN events, bilinguals expressed Path more frequently [χ^2^(1) = 8.0934, *p* = 0.004] but Zero less frequently [χ^2^(1) = 6.5026, *p* = 0.010] than monolinguals. For ACROSS events, bilinguals expressed Manner less frequently [χ^2^(1) = 6.6341, *p* = 0.010] but Zero more frequently [χ^2^(1) = 7.6096, *p* = 0.005] than the monolinguals. Overall, it seems clear that, while the bilinguals generally encoded similar types of spatial information in this locus, they fell slightly short of the monolingual frequency when it comes to encoding Manner or indeed combining it with Path.

### Information in the OTHER Locus in Chinese

The same four categories were established for Chinese. In (21), for example, (additional) Path information is expressed *via* a preposition. In (22), only Manner is expressed in an adverbial without any further information in this locus whereas in (23), the Manner adverbial is combined with a prepositional phrase explicitly marking the Source of motion. In contrast, no such devices are used in (24) and hence “Zero” information expressed in the OTHER locus.


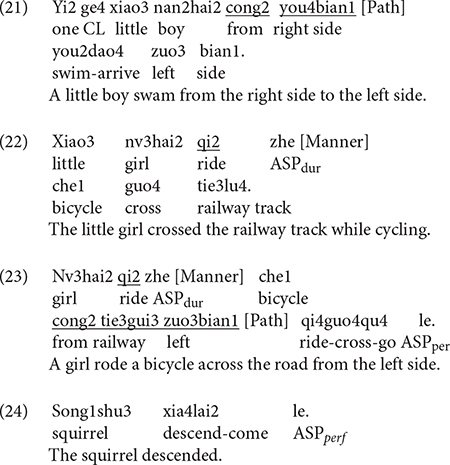


**FIGURE 4:**
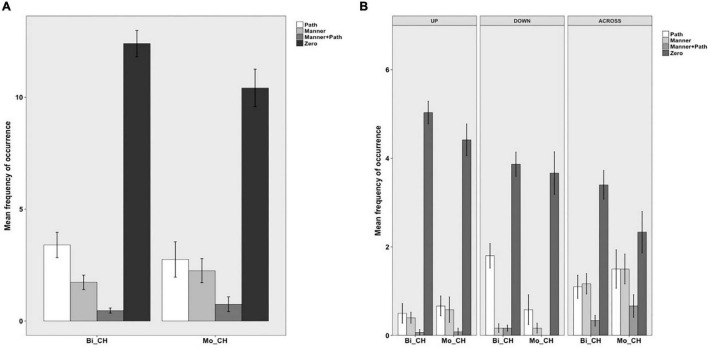
Information in the OTHER locus in Chinese.

Figure 4A below shows information expressed in the OTHER locus in Chinese. As can be seen, both bilinguals and monolinguals predominantly expressed Zero information in this locus, followed by Path and Manner information and very rarely were these two components jointly expressed. The differences among the four categories were significant for both bilinguals [χ^2^(3) = 534.28, *p* < 0.001] and monolinguals [χ^2^(3) = 149.33, *p* < 0.001]. However, a two-way packaging (Path, Manner, Manner + Path, Zero) × group (Bi_CH, Mo_CH) analysis showed no significant interaction, indicating that the distribution of the four patterns did not vary by group. Figure 4B presents information encoded in the OTHER locus in Chinese as a function of path type. A series of two-way packaging (Path, Manner, Manner + Path, Zero) × group (Bi_CH, Mo_CH) analyses on the three path types revealed a significant interaction only for DOWN events due to the bilinguals’ more frequent encoding of Path than monolinguals [χ^2^(3) = 9.8051, *p* = 0.020]. In other words, regardless of path type, bilinguals’ encoding strategies for the OTHER locus fully matched those of the monolinguals.

### Utterance Density in Uyghur

As mentioned earlier, two levels of utterance density were distinguished. UD1 (UD1) if only one component (Path or Manner) was expressed and UD2 if two components were simultaneously expressed (Path and Manner). Both (25) and (26) below illustrate UD2 utterances. Note, however, that, for this measure, the kind of linguistic devices used to encode the components were irrelevant. Thus, in (25), Path is expressed in a case marker while Manner is expressed in the main verb. In (26), Path is in the main verb and Manner in a converb. Note further that, although Path is encoded twice in (26), once *via* case marking and once in the verb, I counted such cases as expressing Path only once. At the constructional level, (25) is satellite-framed and (26) verb-framed (more on this in section “Syntactic Packaging in Uyghur” below).


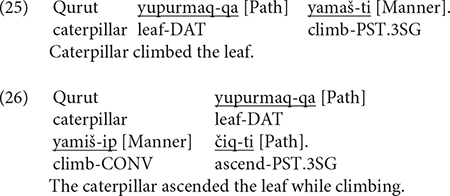


**FIGURE 5:**
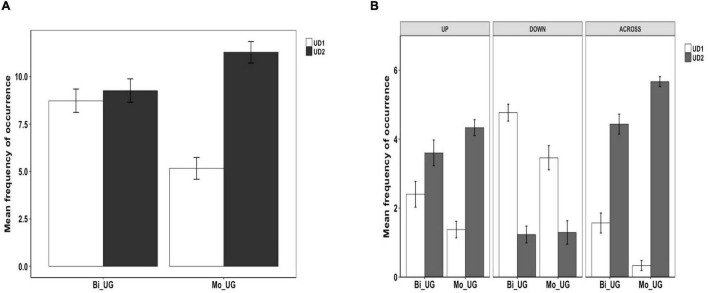
Utterance density in Uyghur.

Figure 5A below shows utterance density in Uyghur across the two groups. The difference between the two categories within the bilingual group was not significant. That is, the bilinguals produced UD1 and UD2 utterances equally frequently, which is unlike the monolinguals, who produced UD2 utterances significantly more frequently than UD1 utterances [χ^2^(1) = 56.045, *p* < 0.001]. A two-way density (UD1, UD2) × group (Bi_UG, Mo_UG) analysis showed significant interaction [χ^2^(1) = 27.957, *p* < 0.001] because the bilinguals produced significantly more UD1 utterances [χ^2^(1) = 6.5326, *p* = 0.010] but less UD2 utterances [χ^2^(1) = 3.9703, *p* = 0.05] compared to monolinguals. Figure 5B presents utterance density for the two groups by path type. A series of two-way density (UD1, UD2) × group (Bi_UG, Mo_UG) analyses on the three path types revealed significant interaction for UP [χ^2^(1) = 9.0633, *p* = 0.002] and ACROSS [χ^2^(1) = 26.65, *p* < 0.001] events due to the significantly higher frequency of UD1 utterances for both path types [χ^2^(1) = 4.0289, *p* = 0.044; χ^2^(1) = 6.889, *p* = 0.008, respectively]. In other words, the bilinguals’ tendency to produce UD1 utterances as observed in the overall analysis stemmed from their descriptions of UP and ACROSS events. Both bilinguals and monolinguals preferred UD1 utterances for DOWN events.

### Utterance Density in Chinese

Example (27) represents a UD1 utterance where only Path information is expressed. Examples (28) to (30) all show UD2 utterance, but as with Uyghur, the components could occur in various linguistic devices. Thus, speakers sometimes used motion verbs that conflate Manner and Path (upward motion) to describe UP events, as in (28). Or typically, they expressed the components in an RVC as in (29). Occasionally, they expressed the two components within an RVC but provided extra Manner information in adverbial devices as in (30). As with the Uyghur data, such instances were counted as expressing Manner only once.


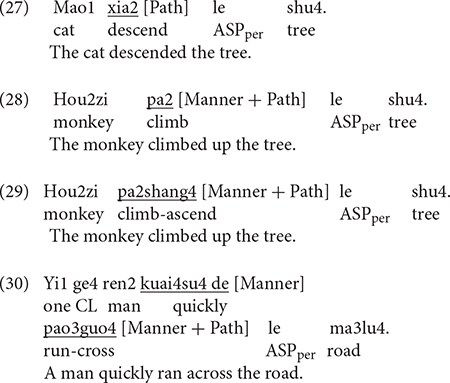


**FIGURE 6:**
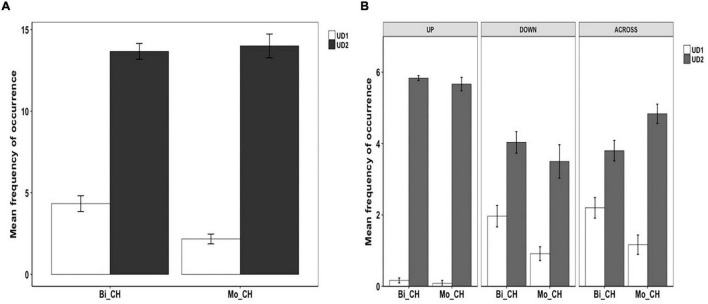
Utterance density in Chinese.

Figure 6A below shows utterance density in Chinese. As we can see, both groups of speakers primarily produced UD2 utterances whereas UD1 utterances were infrequent. The difference between the two density levels was significant for both bilinguals [χ^2^(1) = 152.51, *p* < 0.001] and monolinguals [χ^2^(1) = 116.09, *p* < 0.001]. A two-way density (UD1, UD2) × group (Bi_CH, Mo_CH) analysis revealed significant interaction [χ^2^(1) = 10.446, *p* = 0.001] because the bilinguals produced UD1 responses more frequently than monolingual speakers [χ^2^(1) = 5.2707, *p* = 0.021]. No difference was found between the two groups in the frequency of UD2 utterances. Figure 6B illustrates utterance density in Chinese by path type. A series of two-way density (UD1, UD2) × group (Bi_CH, Mo_CH) analyses on the three path types identified a significant interaction only for ACROSS events [χ^2^(1) = 7.4582, *p* = 0.006] because the bilinguals produced UD1 utterances more frequently than the monolinguals. Overall, the bilinguals fully matched the monolingual preference for UD2 utterances, although they occasionally produced UD1 utterances more frequently than their monolingual counterparts.

### Syntactic Packaging in Uyghur

Recall that this measure focused on how the various linguistic devices are packaged at the syntactic/constructional level. Two categories were distinguished: Tight-Simple if Manner and/or Path are expressed within a single clause and Tight-Complex if they are distributed across two clauses *via* subordination. For example, (31) is a single clause where only Path is expressed. Example (32) is also a single clause but both components are expressed. Example (33) illustrates the Tight-Complex pattern in that Path occurs in the main verb and Manner in the converbial clause.


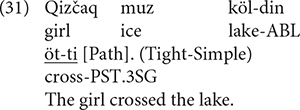



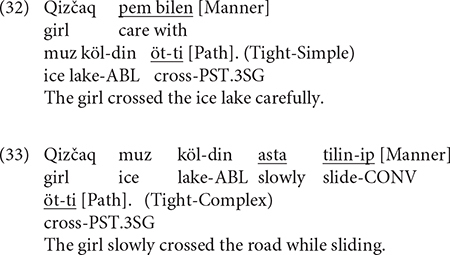


**FIGURE 7:**
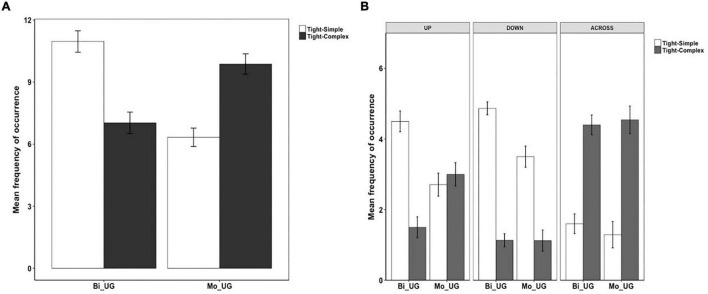
Syntactic packaging in Uyghur.

Figure 7A below presents the patterns of syntactic packaging in Uyghur. On first inspection, it is clear that the bilinguals predominantly used Tight-Simple constructions whereas the monolinguals overwhelmingly used Tight-Complex constructions. The difference between the two categories was significant for both the bilinguals [χ^2^(1) = 25.994, *p* < 0.001] and the monolinguals [χ^2^(1) = 18.724, *p* < 0.001]. A two-way packaging (Tight-Simple, Tight-Complex) × group (Bi_CH, Mo_CH) analysis showed a significant interaction [χ^2^(1) = 43.546, *p* < 0.001] because the bilinguals produced Tight-Simple responses more frequently [χ^2^(1) = 7.6589, *p* = 0.005] but Tight-Complex responses less frequently [χ^2^(1) = 5.726, *p* = 0.016] than monolingual speakers did. Figure 7B displays syntactic packaging in Uyghur by path type. A series of two-way packaging (Tight-Simple, Tight-Complex) × group (Bi_CH, Mo_CH) on the three path type found a significant interaction only for UP events [χ^2^(1) = 25.463, *p* < 0.001] because the bilinguals used the Tight-Simple packaging strategy more frequently [χ^2^(1) = 5.615, *p* = 0.017] but the Tight-Complex strategy less frequently than monolinguals [χ^2^(1) = 5.6106, *p* = 0.017]. That is, the difference between the two groups in the overall analysis was due to their difference for UP events only.

### Syntactic Packaging in Chinese

Example (34) presents a Tight-Simple construction wherein Path and Manner are expressed in an RVC and thus is equipollently framed. Example (35) represents a Tight-Complex construction where Path is expressed in the matrix clause and Manner in the subordinate clause, which is therefore verb-framed.


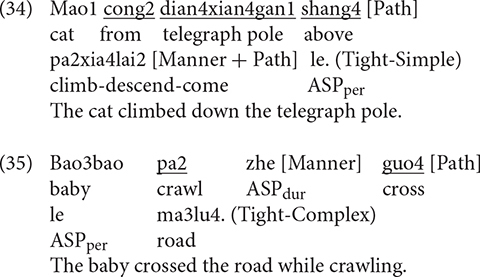


**FIGURE 8:**
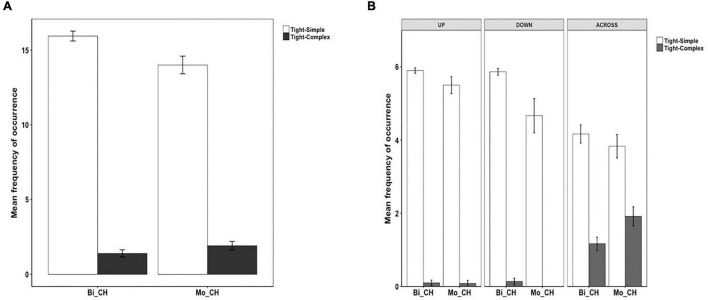
Syntactic packaging in Chinese.

Figure 8A shows the patterns of syntactic packaging in Chinese. As is clear, both groups of speakers showed a strong tendency to use the Tight-Simple strategy, although the Tight-Complex strategy was also used. The difference between the two strategies was significant for both the bilinguals [χ^2^(1) = 429, *p* < 0.001] and the monolinguals [χ^2^(1) = 124.3, *p* < 0.001]. However, a two-way packaging (Tight-Simple, Tight-Complex) × group (Bi_CH, Mo_CH) analysis showed no significant interaction, meaning that bilinguals did not differ from the monolinguals in their use of the two syntactic packaging strategies. Figure 8B illustrates syntactic packaging in Chinese by path type. We see that, across speaker groups and path types, the predominant strategy was Tight-Simple constructions. And while Tight-Complex constructions occurred with all path types, they primarily occurred in relation to ACROSS events. A series of two-way (Tight-Simple, Tight-Complex) × group (Bi_CH, Mo_CH) analyses on the three path types did not find any significant interaction, suggesting that the general pattens observed in the overall analysis applied to the different path types as well.

Our results can be summarized as follows. In terms of information expressed in the verb locus in Uyghur, bilinguals, like monolingual Uyghur speakers, predominantly expressed Path. While both groups of speakers expressed Manner in this locus, particularly with UP and ACROSS events, bilinguals did so more frequently than monolinguals. In Chinese, bilinguals exhibited the same encoding patterns as monolinguals in that they primarily encoded Manner + Path in the verb, which was followed by Path and then Manner. This pattern was consistent across the three path types. In terms of information expressed in the OTHER locus in Uyghur, bilinguals followed the monolinguals in primarily expressing additional Path information *via* case markers and the Manner component, frequently combining it with some sort of Path information (Source or Goal). However, the bilinguals’ frequency of expressing Manner or Manner in combination with Path in this locus fell short of that of the monolinguals, especially for UP events. As to the OTHER locus in Chinese, bilinguals displayed the same tendency as the monolinguals in primarily providing zero spatial information. However, they occasionally offered additional Path information *via* prepositions, Manner or rather infrequently Path and Manner combined.

With respect to utterance density, in Uyghur, bilinguals produced UD1 and UD2 utterances equally frequently. But compared to monolinguals, they produced more UD1 utterances and less UD2 utterances, particularly with UP and ACROSS events. That is, they did not reach the monolingual level of utterance density in their L1 Uyghur. In Chinese, bilinguals, resembling the monolinguals, predominantly produced UD2 utterances, and they did so as frequently as the latter. However, while both groups produced UD1 utterances, the bilinguals produced them more frequently than monolinguals, specifically with ACROSS events. When it comes to the measure of syntactic packaging, the bilinguals, as with monolingual Uyghurs, used both Tight-Simple and Tight-Complex strategies. However, they predominantly used the Tight-Simple strategy, especially for UP events, whereas the monolinguals used the Tight-Complex strategy. In Chinese, the bilinguals followed the monolingual pattern of primarily using the Tight-Simple strategy. Although the Tight-Complex strategy was used by both groups of speakers, particularly in relation to ACROSS events, its use was overall rather infrequent and there was no difference between the bilinguals and the monolinguals.

## Discussion

In this study, I set out to examine whether Uyghur–Chinese adult bilinguals develop language-specific ways of thinking-for-speaking in their two languages. Specifically, I aimed to establish (1) whether and to what extent the bilinguals’ motion event descriptions resembled or diverged from those of the monolingual controls and (2) whether the observed patterns could be accounted for by two influential accounts of CLI, i.e., the structural overlap hypothesis and the co-activation account. Additionally, I hoped to shed light on issues around the longevity of CLI by comparing the current findings with those from Tusun (2019) on Uyghur–Chinese child bilinguals’ motion construal. To this end, Uyghur–Chinese adult bilinguals, Uyghur monolinguals and Chinese monolinguals were invited to describe a set of animated cartoons depicting voluntary motion along three distinct path trajectories (UP, DOWN, ACROSS). Their verbalizations were analyzed in terms of whether Path and Manner components were encoded in the main verb or other devices (Information Locus), how frequently speakers expressed the two key components jointly or alone (Utterance Density) and how the components were packaged syntactically within a motion construction (Syntactic Packaging).

Based on existing research and the two accounts of CLI, I made the following predictions. In Uyghur, the bilinguals would follow the monolingual pattern of expressing Path in the verb and Manner and additional Path information in other devices. As such, they would mostly produce UD2 descriptions expressed in Tight-Complex constructions. In Chinese, although the bilinguals were also expected to largely follow the Chinese equipollent system, i.e., encoding Path and Manner in an RVC, based on the two accounts of CLI, a unidirectional influence was predicted from Uyghur to Chinese, such that they would exhibit a greater tendency for verb-framing, compared to Chinese monolinguals. That is, while the bilinguals would still predominantly produce high-density utterances (UD2), the syntactic packaging strategies they use would be different from the monolinguals in that the former would use Tight-Complex constructions (as characteristic of verb-framing) whereas the latter would use Tight-Simple constructions (as characteristic of equipollent-framing).

My predictions were partially confirmed. Starting with Uyghur, as expected, bilinguals followed the monolinguals in predominantly encoding Path in the verb. That is, bilinguals’ encoding strategy was primarily verb-framed. However, they expressed Manner in the verb significantly more frequently than Uyghur monolinguals, in relation to UP and ACROSS events. This may be an instance of L2 to L1 influence, given that Chinese is also a Manner salient language (cf. Slobin, 2004; Brown, 2015). Yet it is not entirely clear why this CLI only occurred with UP and ACROSS events, but not DOWN events. I shall return to this pattern shortly where I offer a different interpretation. Turning to information expressed in the OTHER locus, my predictions were only partially born out. For instance, bilinguals, like the monolinguals, consistently expressed additional Path information *via* case marking. They also expressed Manner by using converbs. However, they stopped short of the monolingual frequency in terms of combining Path with Manner in this locus, which had cascading effects for measures of Utterance Density and Syntactic Packaging.

Recall that the bilinguals were predicted to primarily produce UD2 utterances, and they would do so using Tight-Complex constructions. Neither of these predictions were born out. For the measure of utterance density, bilinguals produced UD1 and UD2 utterances equally frequently, which was different from the monolinguals who produced significantly more UD2 utterances than UD1 utterances. Cross-group comparisons showed that the bilinguals produced significantly more UD1 utterances but less UD2 utterances than the monolinguals, in relation to UP and ACROSS events. And these differences map onto the measure of Syntactic Packaging in that the bilinguals produced Tight-Simple constructions more frequently but Tight-Complex constructions less frequently than Uyghur monolinguals. The reason for this is likely typological.

As mentioned in section “Motion Event Typology,” in verb-framed languages, simultaneously expressing Path and Manner would typically require more complex syntactic structures (e.g., subordination, adjuncts) that would increase online processing load (cf. Slobin, 2004; Özçalışkan, 2015; Tusun and Hendriks, 2019). And unless Manner is at issue, speakers would typically focus on Path only and hence UD1 utterances in Tight-Simple constructions. That the 10-year-old bilinguals in Tusun (2019) did not reach adult levels of Utterance Density and Syntactic Packaging was attributed to their less developed processing capacities (e.g., working memory) but that even adult bilinguals, who arguably had fully developed processing capacities, did the same, was indeed unexpected. Psycholinguistic research has repeatedly shown that bilingualism imposes greater cognitive demands on the speaker (cf. Michael and Gollan, 2005; Runnqvist et al., 2018). The implication of this for our context seems to be that the typological constraint that verb-framed language speakers generally face, i.e., using syntactically complex structures (e.g., subordination) to produce semantically rich utterances, becomes amplified when the speakers are bilingual. Given that similar observations have been made for Uyghur–Chinese child bilinguals (Tusun, 2019), English-French child bilinguals (Engemann et al., 2012; Engemann, 2016) and Turkish-German/Turkish-French bilinguals (Woerfel, 2018), it is possible that the above-mentioned constraint upon verb-framed language speakers becomes doubly “amplified” when the bilingual is a developing child (see Ji and Hohenstein, 2014 for similar observations in adult L2 acquisition).

As for the results in Chinese, in terms of information in the verb locus, bilinguals fully followed the monolingual pattern of predominantly encoding Path and Manner, followed by encoding Path and occasionally Manner. This general pattern of distribution was not affected by the different Path types in our quantitative analysis. However, a minor qualitative divergence emerged here in that the bilinguals produced 7 instances of Path with UP events when the monolinguals did not, thereby constituting the only instance of L1 to L2 influence in the verb locus. As regards information in the OTHER locus, bilinguals again completely mirrored the monolingual pattern. That is, like the monolinguals, bilinguals tended not to express any spatial information in this locus. But when they occasionally did, they would offer additional Path information *via* prepositions, or Manner information either an adverbial and very rarely Path and Manner together. This general pattern was not affected by the three path types except for the DOWN events where the bilinguals expressed more Path information than monolinguals. This could be the only instance of L1 to L2 influence in the verbal periphery, although this claim, as with the minor qualitative divergence noted for the verb locus, needs to be corroborated by future studies that include a further group of bilingual controls (e.g., Spanish/French-Chinese).

My prediction regarding Utterance Density in Chinese was confirmed. Bilingual speakers, like monolinguals, produced UD1 and UD2 utterances. However, UD2 utterances were the overwhelming majority. Interestingly, bilinguals produced significantly more UD1 utterances than the monolingual controls and this was due to their responses for the ACROSS events. Recall that this was also the case for the bilinguals’ Utterance Density for Uyghur. A qualitative look at the data revealed that in such responses, the bilinguals would typically present the boundary-crossing events as taking place within a general location (e.g., “the boy is swimming in the river”) and only express Manner information (see earlier discussion on Uyghur verb locus). Previous studies on child L1 acquisition (cf. Hickmann et al., 2018; Hendriks et al., 2021) and bilingual acquisition (cf. Tusun, 2019; Engemann, 2021) have documented the same phenomenon and argued that this may be due to the possibility that boundary crossing poses a unique challenge for children’s verbalization because it involves a conceptually more complex type of path configuration, i.e., categorical change of location, as opposed to other events that involve a gradual change of location (e.g., UP, DOWN, TOWARD, AWAY, ALONG). While it is inappropriate to compare adult bilinguals with fully developed cognitive capacities to young developing children, the challenge to conceptualize and verbalize boundary-crossing events during online production may still apply to our bilingual speakers (see Hendriks and Hickmann, 2015 for a similar finding in adult L2 acquisition).

My prediction about Syntactic Packaging was not borne out. Recall that, in light of the structural overlap hypothesis and the co-activation account, I had expected the bilinguals to show a greater reliance on the verb-framed strategy shared by Uyghur and Chinese, which would lead to an increased use of Tight-Complex constructions. Our results showed no such L1 to L2 influence. Rather, the bilinguals systematically adopted the L2-specific equipollent-framing system, which is Tight-Simple and crucially, this pattern was not influenced by the different path types at all. This observation is important because, as seen in Figure 8B, Chinese monolinguals used the verb-framed Tight-Complex construction for all but DOWN events and in fact they used such constructions fairly frequently with ACROSS events. As per the two accounts of CLI, Uyghur to Chinese influence should have been most prominent with ACROSS events, but this is not what we see in the data. Although the difference in Tight-Complex constructions between the two groups did not reach statistical significance for the ACROSS events, that Chinese monolinguals still used them slightly more frequently than the bilinguals is telling, because the bilinguals’ general “avoidance” of Tight-Complex constructions we observed in Uyghur seems to be at play in Chinese as well. And the same processing constraints mentioned earlier on the production of verb-framed constructions could be invoked to account for this (cf. Slobin, 2004; Özçalışkan, 2015).

Thus, our combined results on the bilinguals’ Utterance Density and Syntactic Packaging in L2 Chinese show that crosslinguistic overlap, an important factor for both the structural overlap hypothesis and the co-activation account, does not necessarily lead to CLI. How do we account for this lack of CLI then? In an attempt to offer a unifying account of bilingual language processing, with a particular emphasis on CLI, Filipović and Hawkins (2019) proposed what they called the Complex Adaptive System Principles (CASP) Model. Specifically, these authors contend that bilingual language processing is generally underpinned by a number of principles (i.e., minimizing learning effort, minimize processing effort, maximize expressive power, maximize efficiency in communication, maximize common ground). While “maximize common ground” is also assumed within the structural overlap hypothesis and the co-activation account, the CASP Model additionally posits that the bilingual speaker has to strike a balance between the need for maximizing crosslinguistic structural overlap and other key factors such as processing cost and communicative efficiency. Specifically, they argue that “the most proficient bilingual speakers will also be the most efficient: they know when and how best to make common ground with the least required learning and processing effort while achieving maximum expressive power fit for the specific communicative goals” (ibid., p. 1240).

Now, in the case of Uyghur–Chinese bilinguals, recall that we had instructed the participants to be maximally explicit, i.e., to focus on both Path and Manner, so that an imaginary figure who had no access to the cartoons could reconstruct the events based on the former’s descriptions (see section “Experimental Stimuli and Procedure”). Although the bilinguals could have used the verb-framing strategy as a result of the principles of “maximising common ground” and “maximising communicative efficiency,” given our experimental situation, this would stand in opposition to the principle of “minimising processing effort.” That is, to encode Path and Manner simultaneously in a verb-framed construction, bilinguals would have to use Tight-Complex constructions that would increase the processing load. On the other hand, the L2 Chinese offers a readily available equipollently framed strategy (i.e., RVC) that is Tight-Simple and presumably entails less processing costs. In such a case, our bilinguals seemed to have bypassed the shared structures in their L1 and L2 and opted for the L2-specific strategy that allowed them to achieve maximal communicative efficiency with the least processing effort. Thus, it seems that, while structural overlap is an important factor for CLI to occur, it can be trumped by processing and communicative efficiency concerns during bilingual speech production.

I noted earlier regarding the verb locus in Uyghur that bilinguals used Manner verbs more frequently for UP events (11%) than monolinguals (7%). Indeed, a closer inspection of the Chinese data revealed that the bilinguals displayed the same tendency to use Manner verbs to describe UP events. That is, while monolinguals exclusively used an RVC to encode Manner and Path (e.g., pa2shang4 – “climb-ascend”), bilinguals would occasionally use Manner verbs (e.g., pa4 – “climb up”) with an inherent upward meaning to encode UP events (5%). It is possible that this is another instance of the bilinguals’ maximizing common ground and efficiency in communication. Specifically, when confronted with verbalizing UP events in Uyghur and Chinese, the bilinguals have some choices to make. In Uyghur, to express Path and Manner, one either has to use the canonical verb-framed construction, which is syntactically complex or capitalize on lexical items (i.e., *yamašmaq* – “climb”) that enable them to express both semantic components with less processing effort. Similarly, in Chinese, bilinguals can either express the two components in an RVC or in a single verb that allows the expression of both components. But given that efficiency generally results in a preference for structural and grammatical simplicity and that it can be maximized by using simple forms when this is possible (Filipović and Hawkins, 2019, p. 1229), our bilinguals seem to have occasionally capitalized on options that are not only shared across the two languages (i.e., motion verbs with similar meanings: *yamašmaq* vs. *pa2*) but are also structurally simpler and thus more processing-efficient.

Bilinguals exhibited further qualitative divergences from the monolinguals in that they would sometimes use semantically general motion verbs that were absent in the latter. While this trend existed in both Uyghur and Chinese, it was more pronounced in the latter. Specifically, such instances in Uyghur were limited to ACROSS events where the bilinguals would use the deictic verb *ketmek* – “to go” rather than Path verbs like *ötmek* – “to cross.” As such, boundary-crossing was not explicitly coded, but the Source and/or Goal of motion was indicated as in (34). Parallel cases abound in Chinese. In (35), for example, Manner and Path are expressed in an RVC, but the V2 element therein does not make it clear that the little baby crossed the road. Instead, both the Source and the Goal of the event are provided. Example (36) is a response for a DOWN event, but again, the downward nature of the motion can only be established thanks to the Ground element that follows the RCV. That is, in descriptions such as these, the conformation component of Path (the main geometric schema of a Path) was not overtly expressed but rather implied *via* (a combination of) various Vector components (see Talmy, 2016 for detail). In any event, similar tendencies for bilinguals’ use of semantically general verbs that can be applied in various contexts have been documented in previous studies on child (cf. Álvarez, 2008; Engemann, 2013; Tusun, 2019) and adult bilinguals (cf. Park, 2020) and it has been suggested that it may reflect a more general bilingual strategy for reducing processing costs (cf. Filipović and Hawkins, 2019).


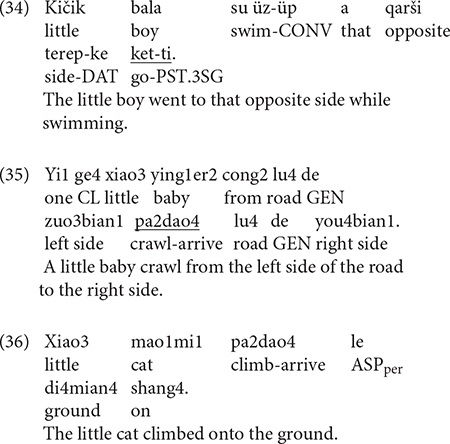


A further qualitative divergence concerns the bilinguals’ use of what can be termed the “atypical constructions” in Chinese that were not attested in the monolinguals. In the whole data set, there were 20 instances of responses as exemplified in (37), where Manner and Path are expressed not in an RVC but a concatenation of two independent verbs without any overt marker indicating the syntactic and semantic relationship between them. Since such descriptions were produced at least once by 14 out of 30 participants, they cannot be considered an outlier phenomenon and curiously, such constructions occurred exclusively with the ACROSS events. I indicated earlier that boundary-crossing events may pose a greater challenge for bilingual speakers and these constructions may be related to this. Indeed, Engemann (2021) also reports that English-French child bilinguals produce ungrammatical motion constructions specific to boundary-crossing events (e.g., *nager* *à travers une rivière “swim across the river”). Engemann attributes such idiosyncrasies to the influence of English framing patterns due to co-activation and it could be that the Chinese atypical constructions also occurred resulting from co-activation of Uyghur framing patterns. But this seems rather unlikely because, unlike English and French who do not share structural overlap (at least for encoding boundary-crossing events), and therefore speakers may be “forced” to opt for ungrammatical constructions, Uyghur and Chinese clearly do, i.e., verb-framing. Instead, recent findings on L2 acquisition of Chinese may shed light on the nature of these atypical constructions.

Several studies on English (cf. Ji and Hohenstein, 2014) and French (cf. Arslangul et al., 2018) L2 learners of Chinese showed that such atypical constructions, as in (38), are highly persistent even at the advanced levels of proficiency. But significantly, the child bilinguals in Tusun (2019) also produced such atypical constructions, exclusively with ACROSS events as well. That is, despite their early age of onset, both Uyghur–Chinese child and adult bilinguals exhibit traits typical of adult L2 acquisition. Recent discussions around sensitive periods in language acquisition suggest that it is such adult-L2-like traits that qualify early successive bilingualism as a kind of L2 acquisition, distinct from simultaneous bilingualism (cf. Meisel, 2018). These discussions have only relied on acquisition data on various aspects of morphosyntax and our data seem to reveal, for the first time, that similar age effects may be at work in the motion domain. Interestingly, in an earlier study, Engemann (2016) reported an absence of age effects in motion expression, based on the observation that English-French successive bilinguals’ descriptions, while often idiosyncratic, did not differ in their framing strategies from the simultaneous bilinguals. What is fundamentally different about the Chinese atypical constructions is that they are not just idiosyncratic but are unacceptable in Chinese grammar (cf. Ji and Hohenstein, 2014). The relatively small number of occurrences of such constructions necessarily limits the wider import of my qualitative observation, but at a minimum, the age effects argued here and their absence in Engemann (2016) highlights the need to examine as many language pairs, preferably typologically more distant ones, so that we can better delineate, insofar as possible, the boundaries in bilingual speech between what is idiosyncratic but still acceptable and what is outright ungrammatical because doing so may have far-reaching implications for our understanding of the nature of bilingual language representation (cf. Meisel, 2018).


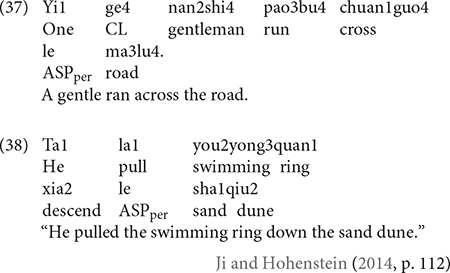


But that these constructions occurred only with ACROSS events merit comment. We noted earlier how boundary-crossing events are problematic for L1 and L2 learners due to their inherent conceptual complexity (e.g., Ji et al., 2011b; Hendriks and Hickmann, 2015; Treffers-Daller and Tidball, 2016; Hickmann et al., 2018; Hendriks et al., 2021). And if the atypical constructions indeed signal potential age effects, then it is possible that such effects are more likely to “surface” when the verbalization process involves the form-meaning mapping of a conceptually complex Path trajectory (i.e., boundary crossing). This hypothesis needs to be tested in future research, but that Uyghur–Chinese child bilinguals displayed the same pattern adds weight to this interpretation. In any case, that these and other qualitative divergences occurred only with ACROSS events extends, for the first time, previous insights about the special status of boundary-crossing to the context of early successive bilingualism.

Finally, I indicated in section “The Case for Studying Motion Event Expressions in Uyghur–Chinese Adult Bilinguals” that a comparison of our findings with those from Tusun (2019) on Uyghur–Chinese early successive child bilinguals would shed light on the issue of the longevity of CLI. To recapitulate, in that study, I investigated how 4-, 6-, 8-, and 10-year-old Uyghur–Chinese early successive bilinguals (with an age of onset of 3;2) acquired motion expressions in Uyghur and Chinese. Two findings therefrom are highly relevant to the present discussion. In terms of their L1 Uyghur, although the child bilinguals followed the target lexicalization pattern of encoding Path in the verb and Manner in the converb from age 4, they stopped short of the adult frequency of combining these two devices to produce UD2 responses even at age 10. Meanwhile, in their L2 Chinese, the bilinguals relied on the verb-framed pattern as a main strategy until age 8 when they eventually overcame L1 influence and fully converged on the target equipollent system. That is, unlike their L1 Uyghur, child bilinguals reached the adult frequency of UD2 responses at age 10, while still producing significantly more UD1 than Chinese adults. Remarkably, this is exactly what the adult bilinguals did as well: in Uyghur, they fell short of the monolingual level of utterance density; in Chinese, they behaved like the monolinguals in predominantly producing UD2 utterances, although like their 10-year-old counterparts, the adult bilinguals produced more UD1 utterances than Chinese monolinguals. Otherwise said, there was no developmental change for Uyghur–Chinese bilinguals from age 10 to adulthood. And given the absence of CLI in 10-year-old bilinguals and in the adult bilinguals, it is clear that there was no increase of CLI, as the co-activation account conceptualized within crosslinguistic priming would predict (cf. Bernolet and Hartsuiker, 2018; Hervé and Serratrice, 2018; Van Gompel and Arai, 2018; Bosch and Unsworth, 2020). Rather, our findings are consistent with the structural overlap hypothesis in that once the target systems are fully established, they operate relatively independently from each other.

Note finally that while the above observation echoes some studies reporting a lack of CLI in motion expression (e.g., Wang and Wei, 2021), it is incompatible with a recent developmental study by Engemann (2021) who reports magnified CLI in older English-French bilinguals than in younger ones. However, this seeming incompatibility could be resolved by invoking language-specific properties if we consider that in Engemann (2021), it was French that was the “vulnerable” language. Specifically, the French motion system has been characterized as rather opaque due to the considerable variability adult French speakers show when expressing motion. This variability/opaqueness in the input has been argued to render the acquisition of motion expressions particularly problematic for L1 (e.g., Engemann et al., 2012; Hickmann et al., 2018; Hendriks et al., 2021) and L2 (e.g., Hendriks et al., 2008; Hendriks and Hickmann, 2015; Engemann, 2016) learners alike. In contrast, the Uyghur–Chinese bilinguals deal with two highly systematic and hence transparent motion systems (Ji et al., 2011a,b,c; Tusun and Hendriks, 2019, in press). It is possible, therefore, that the occurrence and longevity of CLI interacts with language-specific factors such that CLI may be more likely to occur, persist or even increase when the target language presents a variable/opaque system (cf. Hendriks and Hickmann, 2015; Hulk, 2017; Egger et al., 2018; Filipović and Hawkins, 2019).

## Conclusion

In this study, I set out to explore whether and to what extent Uyghur–Chinese adult bilinguals develop language-specific ways of *thinking-for-speaking* in their construal of voluntary motion events, how they compare with the monolingual controls and how two accounts of CLI, i.e., structural overlap (Hulk and Müller, 2000; Hulk, 2017) and co-activation (Nicoladis, 2006, 2012), could account for the observed patterns. To this end, I analyzed motion descriptions based on animated cartoons along several dimensions deemed relevant in motion event typology. Overall, my analyses showed that the bilinguals’ *thinking-for-speaking* patterns were highly language-specific with little CLI. Specifically, bilinguals followed the Uyghur lexicalization pattern of expressing Path in the verb and Manner (when expressed) in a converb. However, they fell short of the monolingual level of simultaneously expressing the two components and of using Tight-Complex constructions, which I accounted for in terms of typological and possible processing constraints. In sharp contrast, bilinguals fully patterned with Chinese monolinguals in all aspects of motion expression, which I attributed to the systematicity of the Chinese motion system and the facilitative effect of some readily accessible structures (i.e., RVC). To shed light on issues around the longevity of CLI, the current findings were compared with previous studies on Uyghur–Chinese child bilinguals (Tusun, 2019). No differences were found between the two groups, indicating that there were no developmental changes either in aspects of motion expression or in the patterns of CLI from 10 years (the oldest child group) into adulthood. As such, our findings lend support to the structural overlap hypothesis which predicts CLI to be a developmental phenomenon.

## Data Availability Statement

The raw data supporting the conclusions of this article will be made available by the authors, without undue reservation.

## Ethics Statement

The studies involving human participants were reviewed and approved by the Ethics Committee of the Department of Theoretical and Applied Linguistics, University of Cambridge. The patients/participants provided their written informed consent to participate in this study.

## Author Contributions

The author confirms being the sole contributor of this work and has approved it for publication.

## Conflict of Interest

The author declares that the research was conducted in the absence of any commercial or financial relationships that could be construed as a potential conflict of interest.

## Publisher’s Note

All claims expressed in this article are solely those of the authors and do not necessarily represent those of their affiliated organizations, or those of the publisher, the editors and the reviewers. Any product that may be evaluated in this article, or claim that may be made by its manufacturer, is not guaranteed or endorsed by the publisher.

## Footnotes

2 The Chinese monolingual data were collected by Ji et al. (2011a) following the same experimental procedure used in this study. I thank them for allowing me to use their data.

3 I thank Henriëtte Hendriks for permission to use the stimuli.

## Appendix

### Appendix 1

Example of an ACROSS event (here shown as a still)

### Appendix 2

List of the stimuli used to elicit motion event descriptions

UP items

1. A mouse climbs up a table.
2. A caterpillar crawls up a plant.
3. A cat jumps up a telephone pole.
4. A bear climbs up a tree.
5. A squirrel runs up a tree.
6. A monkey climbs up a palm tree.

DOWN items

1. A mouse slides down a table.
2. A caterpillar crawls down a plant.
3. A cat jumps down a telephone pole.
4. A bear climbs down a tree.
5. A squirrel runs down a tree.
6. A monkey climbs down a palm tree.

ACROSS items

1. A baby crawls across a street.
2. A man runs across a road.
3. A boy slides across a river.
4. A boy swims across a rover.
5. A girl skates across a lake.
6. A woman cycles across train tracks.
